# Editorial: Insights in protein biochemistry: protein biophysics 2022

**DOI:** 10.3389/fmolb.2023.1207184

**Published:** 2023-04-28

**Authors:** Nikolaos E. Labrou, Hang Fai Kwok, Qi Zhang

**Affiliations:** ^1^ Laboratory of Enzyme Technology, Department of Biotechnology, School of Applied Biology and Biotechnology, Agricultural University of Athens, Athens, Greece; ^2^ Department of Biomedical Sciences, University of Macau, Macau SAR, China; ^3^ Department of Chemistry, Fudan University, Shanghai, China

**Keywords:** atomic force microscopy, biophysics, cryo-electron microscopy (cryo- EM), molecular dynamics, markov random field (MRF), protein docking, replisome, single-molecule fluorescence

**FIGURE 1:**
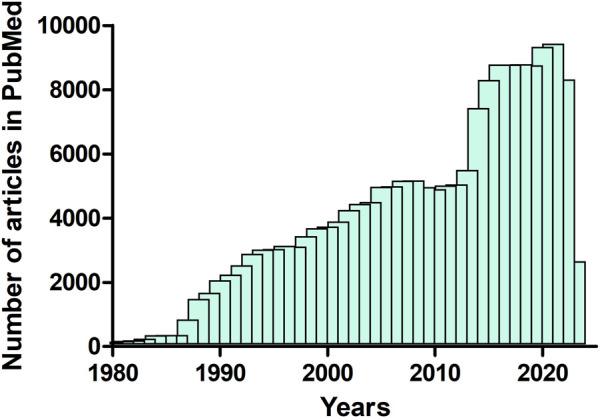
Number of articles (review and research articles) published between 1980 and April 2023 that contain the term “protein biophysics” (data from PubMed, https://pubmed.ncbi.nlm.nih.gov/).

This Research Topic highlights diverse biophysical approaches, methods and tools available for a better understanding of structure-function relationships in proteins. Protein biophysics is crucial for understanding how proteins are formed and function. Proteins are the most complex and versatile molecules known to us. They can be controlled by a broad range of mechanisms, which, in turn can regulate a variety of biological processes (Abdulla et al., 2023; Bryant, 2023; Durham et al., 2023; Elofsson, 2023). Biophysics can exploit and uncover new knowledge on such biological processes that range in scale from the sub-molecular to the systems level. However, optimizing the available methodology and techniques could be challenging. The area of protein biophysics is growing steadily and has become increasingly important. This is emphasized by the increasing number of published articles (review and research articles) on this area. For instance, analysis of the deposited articles in the biomedical literature published between 1980 and 2023, using data from the PUBMED database (https://pubmed.ncbi.nlm.nih.gov/) showed that the publication rate of ‘protein biophysics’ articles has increased exponentially during the past decades (Figure 1).

In the first article, Fuchigami and Takada have applied a flexible-fitting molecular dynamics (MD) simulation method on a molecular motor protein, myosin V. The MD method allowed the authors to obtain atomic-resolution structural information by superimposing a structural model on high-speed atomic force microscopy (HS-AFM) image. The authors investigated whether flexible fitting MD simulation could be used to infer a conformationally different state from an HS-AFM picture. To achieve this goal, the authors have implemented an effective two-step approach. First, they built models of myosin V bound to the actin filament in two conformational states, the “down-up” and “down-down” states. In the next step, for the obtained HS-AFM image of myosin bound to the actin filament, a flexible-fitting MD simulations using the two states were conducted. By comparing the fitting results, the authors were able to infer the conformational and chemical states of myosin V from the AFM image.

The next article, by Alnabati et al., focuses on cryo-electron microscopy (cryo-EM) (Fukuda et al., 2023). They report on the development of a method (MarkovFit) that can perform simultaneous-rigid fitting of protein subunits into medium-to low-resolution cryo-EM maps using Markov random field (MRF). The method starts by using fast Fourier transform (FFT) to search the conformational space for potential positions of subunits and computes scores that quantify the goodness-of-fit between each subunit and the cryo-EM map as well as the interactions between the subunits. In the next step, subunits and their physical interactions are represented using a MRF graph, and the top final conformations undergo structural refinement. MarkovFit evaluates the fit of individual subunits to the map and subunit interactions efficiently in an integrated fashion. The authors evaluated and validated the MarkovFit method using a wide spectrum of experimental datasets and concluded that it performed better than other existing methods.

The Research Topic continues with an interesting review article on replisome by Wilkinson et al. Replisome is a multi-protein complex that contains all the enzymes and necessary components to perform DNA replication. DNA replication is affected by both intrinsic and extrinsic factors, which lead to roadblocks to replication, causing a number of diseases, including cancer (Patel and Kim, 2023). Protein dynamics play an essential role to the molecular pathways that take place in such DNA lesions, including potential damage bypass. Wilkinson et al., discussed three methods that can be used to study protein dynamics during replisome–lesion encounters in replication reactions. The authors emphasise on ensemble biochemical assays, single-molecule fluorescence, and cryo-electron microscopy. Particular focus is given on the two key model DNA replication systems, derived from *Escherichia coli* and *Saccharomyces cerevisiae*. The authors concluded that the combination of these three methods can provide critical biophysical data that can be exploited for visualizing DNA replication and lesion encounter dynamics in real time.

The identification of the quaternary structures of protein complexes that formed within a cell is crucial as they play a key role in many biological processes. Aderinwale et al., have developed a new multiple-chain docking method, Reinforcement Learning for Multimeric protein docking with LZerD (RL-MLZerD) that builds multiple protein complexes using reinforcement learning (RL). Although the method exploits the same approach that has been used by the authors’ in the previously published method (Multi-LZerD) (Esquivel-Rodríguez et al., 2012), however, RL-MLZerD is based on improved RL to perform efficient search of the conformation space. The authors evaluated and assessed the method on a benchmark dataset of protein complexes with three to five chains. RLMLZerD showed better modelling performance than other existing multiple docking methods under different evaluation criteria.

The articles published as part of this Research Topic emphasize significant advancements in several areas of protein biophysics, such as atomic force microscopy, cryo-electron microscopy, flexible-fitting molecular dynamics and protein docking. Protein biophysics is a thriving scientific subject in which experts from a variety of disciplines collaborated to investigate and create new methodology and techniques for understanding how proteins work. As computational performance and structural analyses continue to improve (Agajanian et al., 2023; Sinha et al., 2023), this field will further broaden our understanding of the diverse functions of proteins, with far-reaching implications for a wide spectrum of applications, including medicine and biotechnology.
